# A biobased Schiff base from protocatechualdehyde and its application in flame-retardant, low-smoke epoxy resin systems†

**DOI:** 10.1039/c9ra06574a

**Published:** 2019-09-30

**Authors:** Weiqi Xie, Shiwen Huang, Shumei Liu, Jianqing Zhao

**Affiliations:** School of Materials Science and Engineering, South China University of Technology Guangzhou 510640 P. R. China liusm@scut.edu.cn psjqzhao@scut.edu.cn +86-13611400566 +86-13609724000 +86-13611400566 +86-13609724000; Key Laboratory of Polymer Processing Engineering, Ministry of Education Guangzhou 510640 P. R. China

## Abstract

Herein, a new renewable Schiff base flame retardant 4,4′-((1*E*,1′*E*)-((oxybis(4,1-phenylene))bis(azanylylidene))bis(methanylylidene))bis(benzene-1,2-diol) (PH-ODA) was prepared by the reaction of protocatechualdehyde with 4,4′-diaminodiphenyl ether (ODA). PH-ODA (acting as a carbonization agent) combined with ammonium polyphosphate (APP) were used as intumescent flame retardants for commercial bisphenol A epoxy resin (DGEBA). For the cured epoxy resin containing 7.5% APP and 2.5% PH-ODA, the limiting oxygen index (LOI) reached 29.9% (with the V-0 rating in UL-94 test), and the peak heat release rate and total smoke production were respectively decreased by 88.1% and 68.3%, compared with pure epoxy resin. The enhancement of fire-safety performance was due to PH-ODA/APP promoting the formation of a compact intumescent char structure. It was also found that the synergism between PH-ODA and APP was helpful to enhance the fire resistance of the epoxy matrix. This work provides a facile and sustainable route for synthesizing Schiff base compounds from biomass-derived resources, possessing great potential for application in highly-effective intumescent flame retardants.

## Abstract 2

A novel bio-based Schiff base compound PH-ODA was successfully synthesized and acted as a carbonization agent of IFRs for fire-safe epoxy resins.

## Graphical abstract

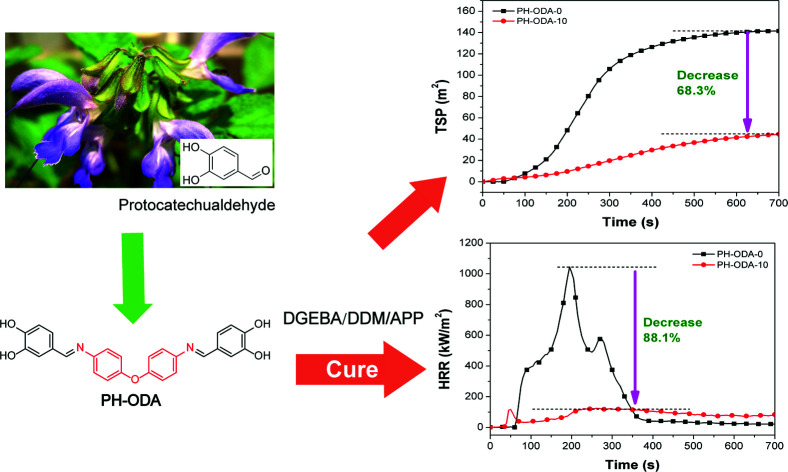

## Introduction

Epoxy resins are a critical class of polymeric materials and have been utilized in various areas (*i.e.*, coatings, adhesives, and laminate materials) for their excellent performance (dimensional stability, satisfactory chemical resistance, good mechanical properties).^1–4^ However, the poor fire safety of epoxy resins is one of the main drawbacks and greatly limits their applications in some fields that require fire resistance.^5–7^

Different types of flame retardants (phosphorus-, nitrogen- and silicon-containing compounds, metal hydroxides and intumescent flame retardants (IFRs)) have been adopted for enhancing the fire-safety performance of epoxy resins.^8–11^ Among them, IFRs are eco-friendly flame retardants with many advantages (*e.g.*, low toxicity and smoke).^12,13^ However, the high addition (more than 25 wt%) of traditional IFRs (such as ammonium polyphosphate and pentaerythritol) is usually required to achieve the fire-safety properties of epoxy resins.^14^ The high addition of IFRs often caused some new problems like the poor mechanical and chemical compatibility performance of the epoxy matrix.^15^ Hence, the fire resistance efficiency of IFRs should be further improved for reducing the adverse impact on other properties of the epoxy thermosets. Recently, many works were reported for solving this problem, and the synthesis of high-performance carbonization agents of IFRs was considered as an important strategy.^16–19^

Schiff base compounds are very important chemicals, which are widely applied for many applications (*i.e.*, drug release, gas separation, and catalysts) because of their diverse properties (*i.e.*, catalytic, magnetic, and biological properties).^20–23^ Recently, the applications of Schiff base compounds for the flame retardant area has attracted much attention due to their good cross-linking charring ability at high temperature.^24,25^ Zhang's work^26^ revealed that the C=N double bonds in Schiff base compounds were able to generate nitrogen-containing hexatomic ring at higher temperature. These hexatomic ring structures make polymers form stable cross-linked networks and endow them with high flame retardancy. Obviously, Schiff base compounds have great potential for the carbonization agents of IFRs. Besides, most of the carbonization agents (*i.e.*, pentaerythritol) used in IFRs are produced from the petroleum-based resource.^27^ In order to meet the urgent needs for alleviating the shortage of fossil resources and achieving the sustainable development, the preparation of bio-based Schiff base carbonization agents for IFRs is a potential and sustainable solution.^28^ Protocatechualdehyde is a plant-derived phenolic aldehyde compound containing two phenolic hydroxyl groups and an aldehyde group, which has been widely used in medicine because of its biological activities.^29,30^ Based on its unique structures and functional groups, protocatechualdehyde might be a promising biomass resource for preparing highly-effective Schiff base flame retardants.

Herein, a novel biorenewable flame retardant 4,4′-((1*E*,1′*E*)-((oxybis(4,1-phenylene))bis(azanylylidene))bis(methanylylidene))bis(benzene-1,2-diol) (PH-ODA) is prepared from renewable protocatechualdehyde and employed as the carbonization agent of IFRs for commercial bisphenol A epoxy resin (DGEBA). It is expected that the flame retardant from a novel biomass-derived aromatic Schiff base compound has outstanding fire resistance efficiency. Moreover, the thermomechanical, thermal and mechanical properties of the cured resins are also evaluated.

## Materials and methods

### Materials

Protocatechualdehyde, 4,4′-diaminodiphenyl ether (ODA), ammonium polyphosphate (APP), and 4,4′-methylenedianiline (DDM) were obtained from Aladdin Reagent Co. Ltd., China. Ethanol, ethyl acetate and acetone were obtained from Guangzhou Chemical Reagent Factory, China. DGEBA (epoxy value = 0.51 mol/100 g) was purchased from SINOPEC Baling company, China.

### Synthesis of Schiff base PH-ODA

In N_2_ atmosphere, protocatechualdehyde (41.4 g, 0.30 mol) was dissolved in 350 mL of ethanol. Then, 4,4′-diaminodiphenyl ether (19.8 g, 0.10 mol) was slowly added into this system. After continuous stirring for 30 min at room temperature, the system was heated to 80 °C and stirred for 6 h. Afterward, the mixture was cooled down and transferred to ice water. The crude product was filtered and washed three times by ethyl acetate to give PH-ODA as a yellow powder (39.0 g, yield 88.7%).

### Preparation of cured epoxy thermosets

The epoxy thermosets were prepared and the formulations of the PH-ODA/APP/DDM/DGEBA system were displayed in Table 1 (the molar ratio of reactive hydrogens to the epoxy groups is 1). Based on the previous works on IFRs system, the selected weight ratio of APP and carbonization agent (PH-ODA) was 3 : 1 for IFRs in this work.^31^ DDM, PH-ODA, and APP were firstly dissolved in acetone (50 °C). Then, the solution was added to a beaker containing DGEBA and stirred for 20 min (50 °C). Afterward, the curing system was transferred in a mould and degassed at 60 °C for 30 min. Finally, the system was cured at 80 °C for 2 h, 110 °C for 1 h, 150 °C for 2 h, and 180 °C for 2 h.

**Table 1:** Stoichiometric formulation of the epoxy system

Samples^a^	DGEBA (wt%)	DDM (wt%)	APP (wt%)	PH-ODA (wt%)
PH-ODA-0	79.83	20.17	0	0
PH-ODA-5	75.84	19.16	3.75	1.25
APP-10	71.85	18.15	10	0
PH-ODA-10	71.85	18.15	7.5	2.5
PH-ODA-15	67.86	17.14	11.25	3.75

a Sample name: PH-ODA-*X*, *X* represents the mass fraction of the sum of PH-ODA and APP in curing system. APP-10 represents 10 wt% of APP in curing system.

### Characterizations

^1^H and ^13^C nuclear magnetic resonance (NMR) spectra were collected with a Bruker NMR spectrometer (Billerica, MA, USA) and deuterated dimethylsulfoxide (DMSO-d_6_) was used as the solvent. The infrared spectra (FT-IR) were obtained with a Vertex 70 spectrometer (Bruker, Billerica, MA, USA) using KBr pellets. Mass spectrometry (MS) spectra were collected with a maXis impact mass spectrometer (Bruker).

Thermogravimetric analyses (TGA) was conducted using a TG-209F1 TGA (Netzsch, Selb, Germany) at a heating rate of 10 °C min^−1^ (N_2_ atmosphere), and the temperature range is from 50 to 700 °C. Dynamic mechanical analysis (DMA) was conducted using a TA instrument (DMA Q800, America) at a heating rate of 3 °C min^−1^ (from 25 to 230 °C). The dimensions of cured samples for measurement were 40 × 10 × 3.0 mm.

Tensile and flexural properties were analyzed based on ASTM D638-08 and ASTM D790-07, respectively, on an Instron-5967 universal electronic testing machine.

UL-94 vertical burning tests were conducted with a UL 94 flame chamber (Fire Testing Technology, UK) according to ASTM D3801-10 (sample dimension of 125 × 13 × 3 mm). Limiting oxygen index (LOI) tests were conducted using an oxygen index instrument (Fire Testing Technology, UK) according to ASTM D2863-97 (sample dimension of 150 × 6.5 × 3.2 mm). Cone calorimeter tests (CCT) were conducted using a FTT cone calorimeter according to ISO5660 (sample dimension of 100 × 100 × 5 mm).

Scanning electron microscopy (SEM) experiments were conducted with a NOVA NANOSEM 430 machine. The sample was sputter-coated with gold before testing. X-ray photoelectron spectrum (XPS) was conducted using an Axis Ultra spectrometer (Kratos, England). Thermogravimetry-Fourier transform infrared spectrometer (TGA-FTIR) tests were conducted with a STA449C/3MFC/G instrument (Bruker, USA) (N_2_ atmosphere, heating rate = 20 °C min^−1^).

## Results and discussion

### Synthesis of PH-ODA

**Fig. 1:**
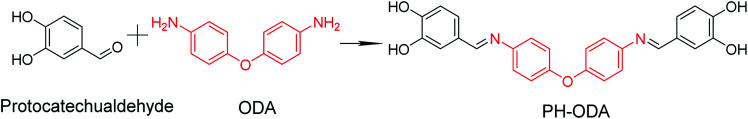
Synthesis route for PH-ODA.

The bio-based aromatic Schiff base compound PH-ODA is synthesized from protocatechualdehyde and ODA in one step depicted in Fig. 1. The synthesis method is facile and high-yield, which provides a feasible way for the scalable and sustainable production of bio-based flame retardant. The structure of the bio-based PH-ODA is characterized using ^1^H-NMR, ^13^C-NMR and HRESI-MS techniques (Fig. S1†). Fig. S1a† displays the ^1^H-NMR spectrum of PH-ODA, and the peak at 9.58 and 9.30 ppm are attributed to –OH. (H_1_ and H_2_), the peak at 8.39 ppm is attributed to –CH=N (H_6_), the signal of the aromatic protons (H_3–5_, H_7_, H_8_) is found at 7.40–6.83 ppm. In the ^13^C-NMR spectrum of PH-ODA (Fig. S1b†), the expected chemical shifts of C atoms are in good agreement with the actual chemical shifts. Fig. S1c† shows an [M + H^+^] ion peak at *m*/*z* 441.14 (molecular formula = C_26_H_20_N_2_O_5_). All these results verify that PH-ODA with a designed structure has been successfully synthesized. The thermal stability of PH-ODA is assessed by TGA (N_2_ atmosphere). As shown in Fig. S1d,† the initial decomposition temperature (*T*_5%_, temperature at 5% weight loss) of PH-ODA is 256.2 °C, and PH-ODA presents very high residue (60.1%) at 700 °C, suggesting an excellent charring ability of PH-ODA.

### Flame-retarded properties

Vertical burning (UL-94) and LOI tests are utilized for evaluating the fire resistance properties (Table 2).^32^ The pure epoxy resin PH-ODA-0 displays the LOI value of 23.5% with melt-dripping and no rating in UL-94 test. 5.0 wt% PH-ODA/APP increases the LOI value to 27.8% without any dripping. By only adding 10 wt% APP to epoxy resin, the LOI value of sample APP-10 reaches 29.1% with no rating in UL-94 test. The fire-safety of epoxy resins is further improved by replacing APP with PH-ODA/APP, and 10.0 wt% PH-ODA/APP (sample PH-ODA-10) increases LOI value to 29.9% with V-0 rating of UL-94 test. The above results reveal that PH-ODA/APP exhibits excellent efficiency on flame-retarded performance, and PH-ODA has synergistic effect with APP in enhancing the fire-safety performance.

**Table 2:** Results of UL-94 and LOI tests for cured epoxy resin

Samples	LOI (%)	*t*_1_ + *t*_2_ (s)	Dripping	UL-94 rating
PH-ODA-0	23.5	Last burning	Yes	No rating
PH-ODA-5	27.8	Last burning	No	No rating
APP-10	29.1	85.8 ± 9.5	No	No rating
PH-ODA-10	29.9	1.1 ± 0.6	No	V-0
PH-ODA-15	32.3	0.5 ± 0.3	No	V-0

**Fig. 2:**
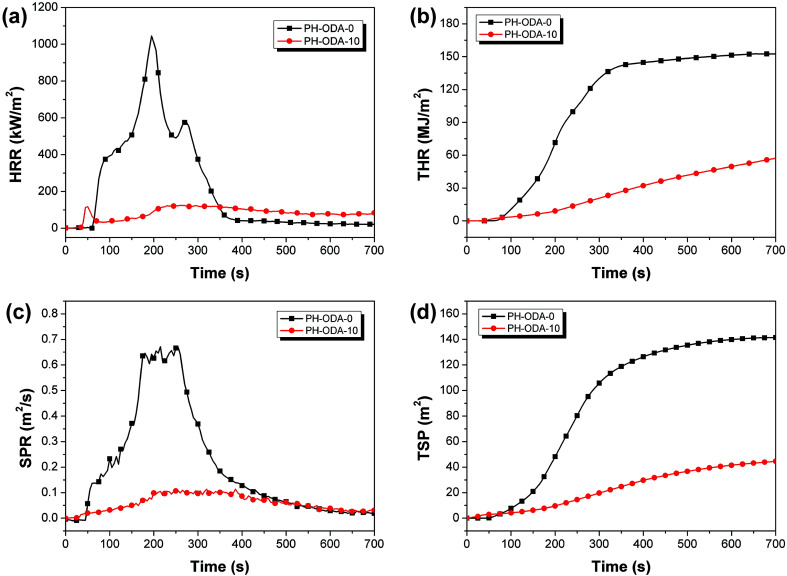
(a) HRR, (b) THR, (c) SPR, and (d) TSP curves of PH-ODA-0 and PH-ODA-10.

The fire-safety of the epoxy thermosets is further assessed with cone calorimeter test (CCT) (Fig. 2 and Table 3).^33^ As seen, the time to ignition (TTI) value of PH-ODA-10 is much lower than PH-ODA-0, which is mainly caused by the degradation of IFRs at the early stage.^24^ The peak heat release rate (pHRR) of PH-ODA-10 is decreased to 124.3 kW m^2^, which is 88.1% lower than PH-ODA-0 (1045.2 kW m^2^). Similarly, compared with PH-ODA-0, PH-ODA-10 shows a 62.3% reduction in total heat release (THR) value. The low HRR and THR values further demonstrate the excellent flame-retarded performance of IFRs. The smoke released from combustion is deemed as an extremely important parameter for fire safety of epoxy resins.^34^ In Fig. 2c and d, compared to PH-ODA-0, the peak smoke production rate (pSPR) and total smoke production (TSP) values of PH-ODA-10 are decreased significantly by 83.1% and 68.3%, demonstrating that the present IFRs are very beneficial to the smoke suppression of epoxy resins. Table S1† lists the reported Schiff base structures for flame retardant epoxy resins. As seen, most of the reported Schiff base compounds contain P element, because they are usually synthesized from the reaction of Schiff base intermediates with DOPO or DPPA, which lead to the high molecular weights of these Schiff base compounds. In this work, the flame retardancy of PH-ODA is attributed to the good cross-linking charring ability of C=N double bond (without introducing DOPO or DPPA), which shows a relatively lower molecular weight compared with the reported Schiff base compounds and thus reduces the steric hindrance of PH-ODA molecule. In addition, the four active phenolic groups make PH-ODA have good compatibility with epoxy resin.

**Table 3:** Cone calorimetry data for cured epoxy resin

Samples	TTI (s)	pHRR (kW m^2^)	THR (MJ m^2^)	SPR (m^2^ s^−1^)	TSP (m^2^)
PH-ODA-0	50	1045.2	152.3	0.65	141.2
PH-ODA-10	25	124.3	57.3	0.11	44.8

### Char analysis

Fig. S2† depicts the photos of the char residues after CCT. It is revealed that the char residue of PH-ODA-0 is fragile and only a little char remains due to its poor charring property. In contrast, the PH-ODA-10 possesses highly compact and intumescent char residues. The observation from SEM (Fig. S3†) shows that the char residue of PH-ODA-0 possesses the cracked and discontinuous char residue with many open holes. For the char residue of PH-ODA-10, many tunnels and a more homogeneous and continuous char residue is generated, which provides a protective barrier for isolating the gases and heat from the epoxy matrix.

**Fig. 3:**
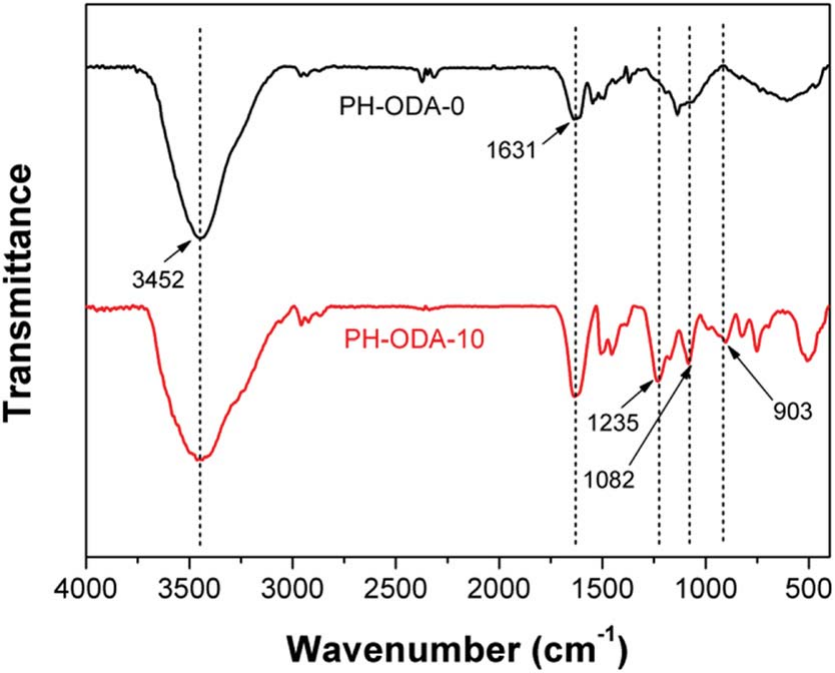
FT-IR spectra of char residues after CCT.

FTIR technique is utilized for analyzing the chemical compositions of char residues after CCT. For char residues of both PH-ODA-0 and PH-ODA-10 in Fig. 3, the peak appeared at 3452 cm^−1^ (N–H and O–H) indicate that amino- and hydroxyl-containing substances exist in PH-ODA-0 and PH-ODA-10 after combustion. The peak at 1631 cm^−1^ is assigned to the stretching vibrations of carbonized compounds. Meanwhile, for PH-ODA-10, several new absorption peaks appear, which are assigned to the phosphorus-containing bands: 1082 cm^−1^ (P=O), 903 cm^−1^ (P–O–C) and 1235 cm^−1^ (P–O–C).^35^ The FTIR results indicate that the nitrogen and phosphorus-containing substances are left after CCT, which jointly promote the formation of intumescent char during the combustion of epoxy matrix. XPS technique is also utilized for analyzing the element composition of the char residues, as listed in Table 4. The char residue of PH-ODA-0 is composed of C, O and N elements and contains no P element. Meanwhile, the P content of the PH-ODA-10 char residue reaches 2.70 wt%, indicating that the phosphorus-containing substances generated from PH-ODA-10 play a very important role in forming the intumescent char, which agrees with the results of FT-IR.

**Table 4:** XPS analysis of char residues after CCT

Samples	C (wt%)	O (wt%)	N (wt%)	P (wt%)
PH-ODA-0	80.87	14.92	4.21	0
PH-ODA-10	85.75	9.82	1.73	2.70

Raman spectroscopy is applied for assessing the graphitization of char by using the ratio of the integrated intensities of D to G bands on Raman spectra. Fig. S4† illustrates the Raman spectra of char residues for PH-ODA-0 and PH-ODA-10, both of which show two distinctive bands *i.e.* D band (1345 cm^−1^) and G band (1591 cm^−1^). D band is related to the disordered carbon structure and G band is related to the stretching vibration of carbon atoms in the crystalline part of graphite layers.^36^ Thus, the ratio of integrated intensities (*I*_D_/*I*_G_) is an indicator of the degree of graphitization. The *I*_D_/*I*_G_ values of PH-ODA-0 char (3.46) is much higher than that of PH-ODA-10 char (3.10). The lower *I*_D_/*I*_G_ value of PH-ODA-10 suggests that the char of PH-ODA-10 possess higher degree of graphitization, indicating that the IFRs (PH-ODA/APP blends) are helpful to enhance the thermal stability and flame retardancy of epoxy composite.^37^

### Thermogravimetric analysis

**Fig. 4:**
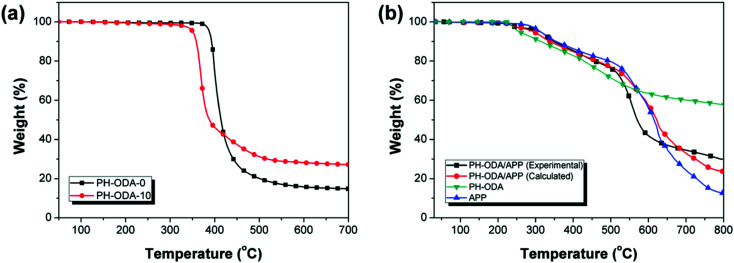
TGA curves of (a) PH-ODA-0 and PH-ODA-10 in N_2_ and (b) experimental and calculated PH-ODA/APP blends in N_2_.

Fig. 4 shows TGA curves of PH-ODA-0 and PH-ODA-10 (N_2_ atmosphere). It is seen that PH-ODA-0 and PH-ODA-10 possess the single weight loss process. In Fig. 4a, PH-ODA-10 starts to degrade at a lower temperature (*T*_d 5%_ = 350.7 °C) than PH-ODA-0 (388.2 °C), and degrades more quickly in the initial degradation stage (350–420 °C), indicating that the early degradation of IFRs facilitates the degradation of epoxy resin, which agrees with the TTI results. When the temperature exceeds 420 °C, the residue of PH-ODA-10 is larger than that of PH-ODA-0, suggesting that the early degraded IFRs stabilizes the residue at higher temperature. Moreover, the residue (700 °C) of PH-ODA-10 is increased to 27.1% from 14.8%. The results are in accordance with the SEM results, suggesting that IFRs promote the char forming during the thermal degradation process and enhance cross-linking reactions in epoxy composites at higher temperature range, improving the thermal stability and char residue of epoxy composites.

The interaction between PH-ODA and APP is verified by the comparison of the calculated and experimental results of PH-ODA/APP mixture during the thermal degradation (Fig. 4b). The calculated curve is achieved based on the combination of the two separate TGA curves of PH-ODA and APP.^38^ It can be seen that the experimental curve is above the calculated curve after 680 °C, suggesting that the reaction between PH-ODA and APP improves the thermal stability and charring properties. In addition, the residue of the experimental curve at 800 °C (30.0%) is higher than the calculated residue (23.7%). The above results further verify the synergistic effect in the IFRs system, which promote the char formation of the epoxy composite.

### TG-FTIR analysis of gaseous phase

**Fig. 5:**
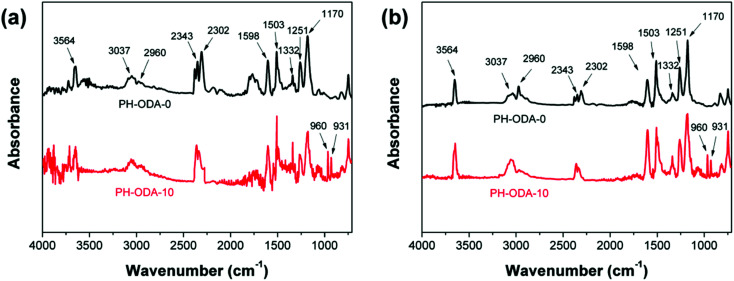
The FTIR spectra of pyrolysis products of PH-ODA-10 and PH-ODA-0 at (a) the initial and (b) maximum degradation temperatures.

TG-FTIR technique is used to investigate the flame-retardant mechanism by detecting the pyrolysis gas generated from the degradation of PH-ODA-0 and PH-ODA-10. According to the TGA result, both PH-ODA-0 and PH-ODA-10 possess the single degradation process. The FTIR spectra at the initial (corresponding to 350 °C for PH-ODA-10 and 390 °C for PH-ODA-0) and maximum (corresponding to 370 °C for PH-ODA-10 and 405 °C for PH-ODA-0) degradation temperatures are illustrated in Fig. 5. The pyrolysis products of both PH-ODA-0 and PH-ODA-10 are analyzed as follow: 3564 cm^−1^ (phenol derivatives), 3037 and 2960 cm^−1^ (aliphatic hydrocarbons), 2343 and 2302 cm^−1^ (CO_2_), 1598, 1503, and 1332 cm^−1^ (aromatic substances), 1251 and 1170 cm^−1^ (ester and ether compounds).^35,39^ Compared with PH-ODA-0, two new peaks occur in the spectrum of PH-ODA-10, which are assigned to the NH_3_ (931 and 960 cm^−1^).^40^ The results confirm that the nitrogen-containing gaseous products from the IFRs in PH-ODA-10 appear in the pyrolysis gas, which has a positive effect on the fire resistance of epoxy resin in vapor phase.

### FTIR analysis of condensed-phase

**Fig. 6:**
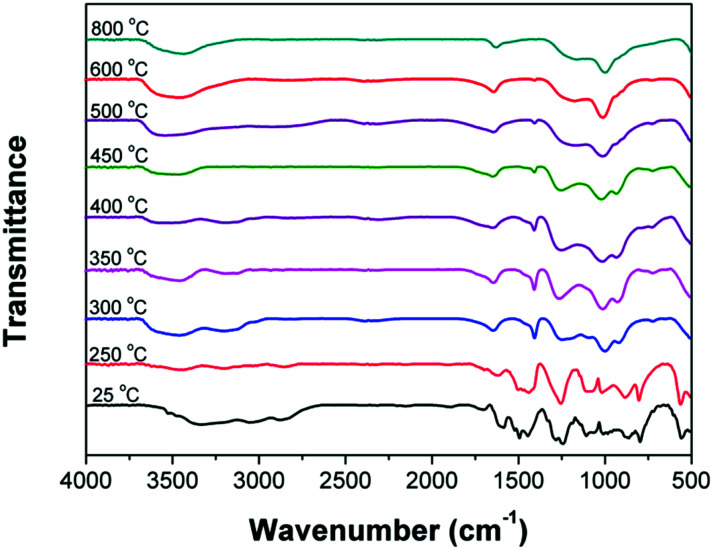
FTIR spectra of the residues of IFRs (PH-ODA/APP blends) heated at different temperatures.

In order to investigate the flame-retardant mechanism of IFRs (PH-ODA/APP mixture) in condense phase, the residues of PH-ODA/APP mixture (at different temperature) were detected by FTIR (Fig. 6). As seen, compared with the spectrum at 25 °C, the peaks at 1585 cm^−1^ and 1437 cm^−1^ (NH_4_^+^ stretching) disappear after 300 °C, which is due to the removal of NH_3_ and agrees with the TG-FTIR results. In addition, the peak of PH-ODA at 3335 cm^−1^ (–OH stretching) disappear and peaks at 1253 and 914 cm^−1^ (P–O–C stretching) appear after 300 °C, which is attributed to the dehydration reaction of PH-ODA with polyphosphoric acid. Besides, the peak at 1001 cm^−1^ (P=O stretching) is found over the whole temperature scope. The above results show that P–O–C and P=O promote the formation of char layer during the combustion of epoxy matrix. The peak appeared at 1650 cm^−1^ (–C=C– stretching) after 300 °C indicates the generation of carbonized substances. Moreover, the peak of PH-ODA at 1487 cm^−1^ (–C=N– stretching) disappear and peak at 1401 cm^−1^ (–C–N– stretching) appear at 300 °C, indicating the formation of amine nitrogen (–C–N–). According to the literature,^26^ the amine nitrogen (–C–N–) converts to nitrogen-containing hexatomic ring after further increasing the temperature. The above results indicate that the reaction of APP with PH-ODA and the self-crosslinking of –C=N– in PH-ODA jointly promote the char forming of the stable char layer.

### Thermomechanical and mechanical properties

**Fig. 7:**
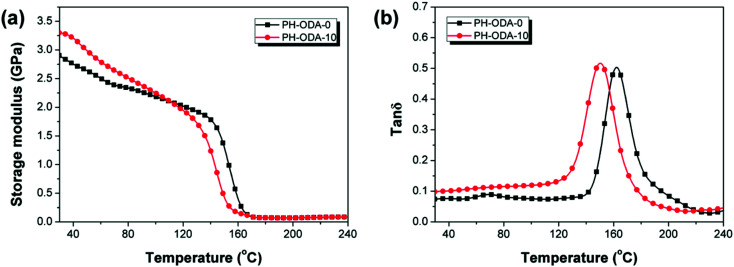
(a) Storage modulus (*E*′) and (b) tan *δ* curves for PH-ODA-0 and PH-ODA-10.

DMA is applied for the evaluation of thermomechanical properties of cured epoxy thermosets (Fig. 7 and Table 5). PH-ODA-10 shows higher storage modulus (*E*′) value (3.29 GPa) than PH-ODA-0 (2.97 GPa) at room temperature, suggesting that the rigid structure of IFRs enhances the stiffness of epoxy thermosets. Besides, PH-ODA-10 possesses a *T*_g_ of 159.5 °C, which is slightly lower than PH-ODA-0 (162.2 °C). Although the four phenolic groups in PH-ODA can react with the epoxy groups, which makes the PH-ODA have better compatibility with epoxy resin. However, the poor interfacial adhesion between the APP (main component in IFRs) and epoxy matrix may lead to the decrease in *T*_g_, which is in accordance with the results in the literature.^41^

**Table 5:** Key parameters collected from DMA for PH-ODA-0 and PH-ODA-10

Samples	*E*′ at 30 °C (GPa)	*T*_g_ (°C)	*ν*_e_ (10^3^ mol m^−3^)
PH-ODA-0	2.97	162.1	5.04
PH-ODA-10	3.29	153.3	4.95

The mechanical properties of PH-ODA-0 and PH-ODA-10 are further evaluated (see Table 6). Compared to PH-ODA-0, the tensile and flexural strengths of PH-ODA-10 are only decreased by 5.1% and 4.2%, respectively. These differences are mainly originated from the lower cross-link density of PH-ODA-10. Meanwhile, the tensile and flexural moduli of PH-ODA-10 are respectively increased by 3.0% and 7.7%, which is mainly a result of the more rigid structures (Schiff base and aromatic structure) in PH-ODA-10.

**Table 6:** Tensile and flexural properties of cured epoxy resin

Samples	Tensile modulus (GPa)	Tensile strength (MPa)	Flexural modulus (GPa)	Flexural strength (MPa)
PH-ODA-0	3.32 ± 0.23	72.8 ± 1.0	2.71 ± 0.11	108.2 ± 5.0
PH-ODA-10	3.42 ± 0.12	69.1 ± 0.9	2.92 ± 0.23	103.7 ± 1.6

## Conclusions

In summary, a novel bio-based Schiff base compound PH-ODA was successfully synthesized and acted as a carbonization agent of IFRs for fire-safe epoxy resins. Only 10 wt% IFRs (2.5 wt% PH-ODA and 7.5 wt% APP) were sufficient to pass UL-94 V-0 rating. The pHRR and THR of PH-ODA-10 were respectively 88.1% and 62.3% lower than those of pure epoxy resins. Similarly, the pSPR and TSP values of PH-ODA-10 were decreased by 83.1% and 68.3%. The enhancement of fire-safety performance was attributed to that PH-ODA/APP promoted the formation of more compact intumescent char structure. This work provides an effective strategy to prepare bio-based carbonization agent of IFRs for epoxy thermosets.

## Conflicts of interest

The authors declare that there are no conflicts of interest.

## Supplementary Material

RA-009-C9RA06574A-s001
