# Supplementary material for: A biobased Schiff base from protocatechualdehyde and its application in flame-retardant, low-smoke epoxy resin systems
Source: RSC Adv. 2019 Sep 30;9(53):30815–22. doi: 10.1039/c9ra06574a (PMC9072551; doi:10.1039/c9ra06574a)
Supplement: RA-009-C9RA06574A-s001 [file RA-009-C9RA06574A-s001.pdf]

Electronic Supplementary Material (ESI) for RSC Advances.
This journal is © The Royal Society of Chemistry 2019

Supporting information

A biobased Schiff base from protocatechualdehyde and its application in flame-retardant, low-smoke epoxy resin systems

Weiqi Xie[a], Shiwen Huang[a], Shumei Liu[ab], and Jianqing Zhao*[ab]

[a]School of Materials Science and Engineering, South China University of Technology, Guangzhou, 510640, P. R. China

[b]Key Laboratory of Polymer Processing Engineering, Ministry of Education, Guangzhou 510640, P. R. China

Corresponding authors

*Tel./Fax: +86-13609724000. E-mail: psjqzhao@scut.edu.cn (Jianqing Zhao)

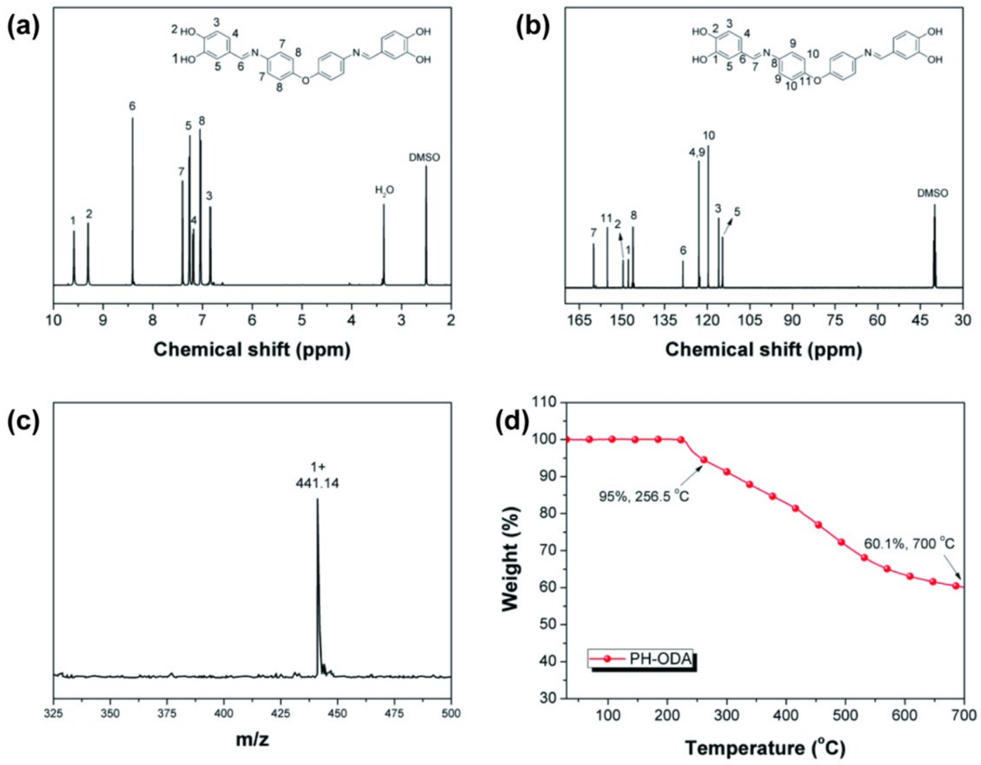

Fig. S1 (a) ^{1}H-NMR, **(b)** ^{13}C-NMR, **(c)** HRESI-MS spectrum, and **(d)** TGA curve of PH-ODA

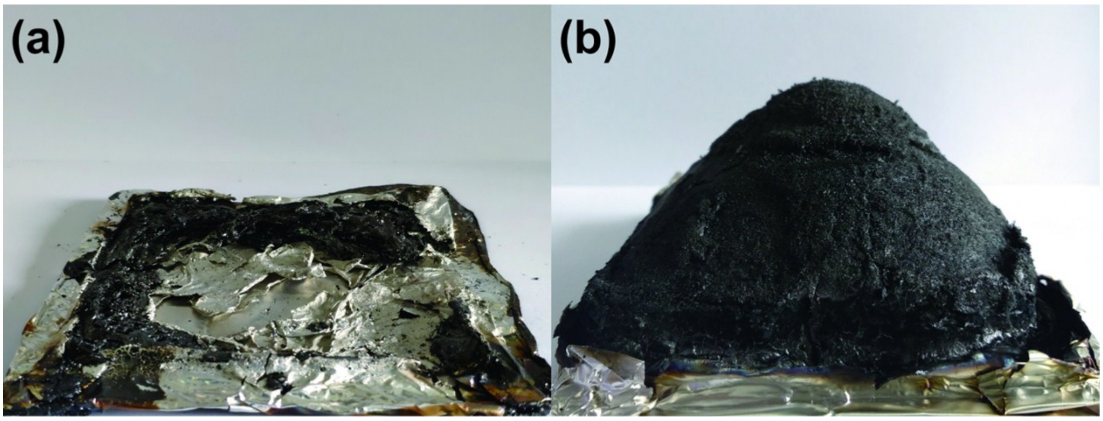

Fig. S2 Photos of char residual of **(a)** PH-ODA-0 and **(b)** PH-ODA-10 after CCT

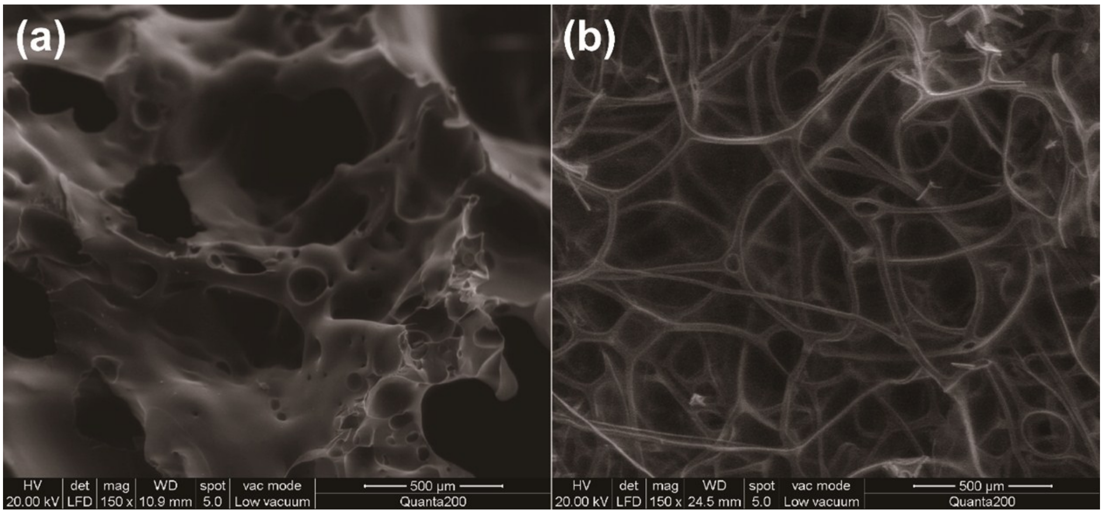

Fig. S3 SEM images of char residual of **(a)** PH-ODA-0 and **(b)** PH-ODA-10 after CCT

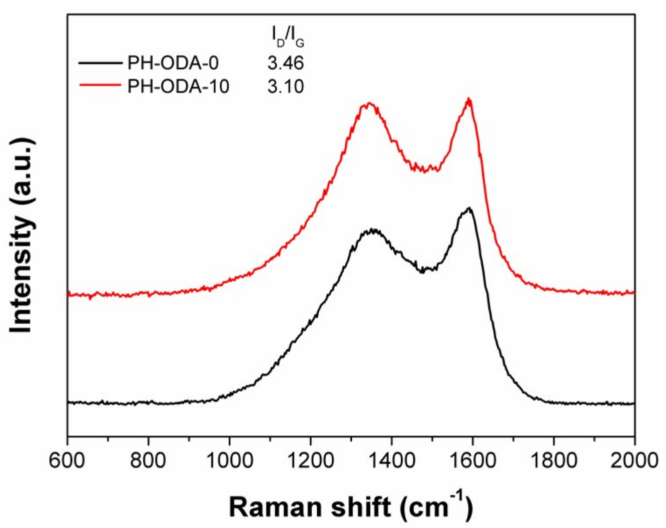

Fig. S4 Raman spectra of char residues for PH-ODA-0 and PH-ODA-10 after CCT

Table S1 List of the reported Schiff base structures for flame retardant epoxy resins

Schiff base compound	Chemical structure	References
PH-ODA	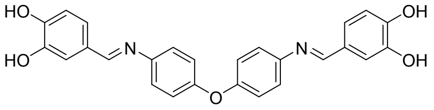	This work
DP-DDE	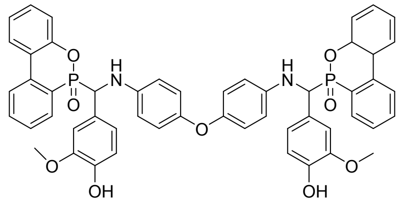	[S1]
DP-DDS	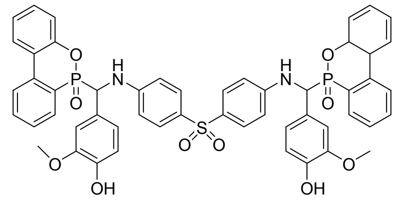	[S1]
D-bp	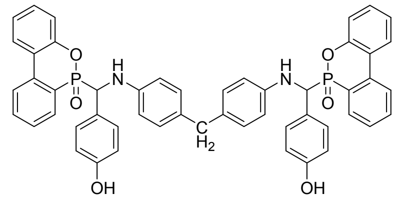	[S2]
P-MSB	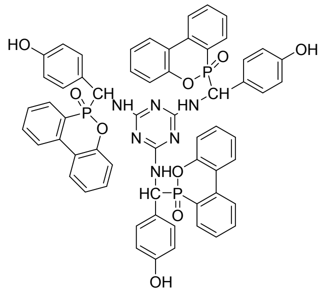	[S3]
HD-DPPA	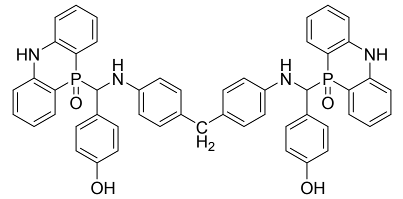	[S4]
H-DPPA	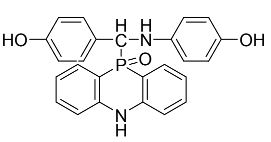	[S5]
DPN	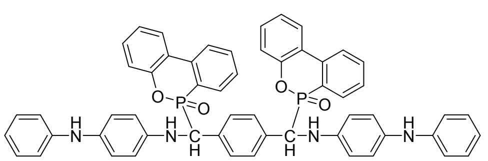	[S6]

References

S1. Gu, L., Chen, G., & Yao, Y. (2014). Polymer degradation and stability, 108, 68-75.

S2. Xu, W., Wirasaputra, A., Liu, S., Yuan, Y., & Zhao, J. (2015). Polymer degradation and stability, 122, 44-51.

S3. Xiong, Y., Jiang, Z., Xie, Y., Zhang, X., & Xu, W. (2013). Journal of Applied Polymer Science, 127(6), 4352-4358.

S4. Luo, Q., Yuan, Y., Dong, C., Liu, S., & Zhao, J. (2016). Materials Letters, 169, 103-106.

S5. Luo, Q., Yuan, Y., Dong, C., Huang, H., Liu, S., & Zhao, J. (2016). Industrial & Engineering Chemistry Research, 55(41), 10880-10888.

S6. Chen, T., Chen, X., Wang, M., Hou, P., Jie, C., Li, J., ... & Dai, L. (2018). Polymers for Advanced Technologies, 29(1), 603-611.
